# Sex and gender considerations in cross-cultural traumatic stress studies

**DOI:** 10.1080/20008066.2024.2408194

**Published:** 2024-10-15

**Authors:** Rachel Langevin, Sophie Beaudette, Dany Laure Wadji, Sara Abou Chabake, Carolina Gonzalez, Dan Jenkins, Safa Kemal Kaptan, Jessica Lambert, Tilahun Belete Mossie, Rosario Spencer

**Affiliations:** a Department of Educational and Counselling Psychology, McGill University, Montreal, Canada; b Department of Psychology, Université de Montréal, Montreal, Canada; c School of Psychology and Wellbeing, University of Southern Queensland, Ipswich, Australia; d Institute for Life Course Health Research, Stellenbosch University Tygerberg Campus, Cape Town, South Africa; e Department of Psychology, Boğaziçi University Bebek, İstanbul, Türkiye; f International Programmes, DIGNITY, Copenhagen, Denmark; g Department of Psychiatry, College of Medicine and Health Sciences, Bahir Dar University, Bahir Dar city, Ethiopia; h Faculty of Psychology, Talca University, Talca, Chile

**Keywords:** Post-traumatic stress, culture, cross-cultural studies, sex, gender, research methodologies, Estrés postraumático, cultura, estudios transculturales, sexo, género, metodologías de investigación

## Abstract

Following the 1st Conference of the Global Collaboration on Traumatic Stress, the consortium committed to systematically integrating sex and gender considerations in their endeavours, which aligns with the *European Journal of Psychotraumatology*'s Gender Policy. This initiative is vital for understanding trauma's complex impacts, but also presents significant challenges in cross-cultural research. This letter, co-authored by researchers from across the globe, outlines these challenges and proposes mitigation strategies. First, definitions of sex and gender are provided from a Western perspective, while acknowledging cultural differences in these concepts. Second, the relevance of integrating sex and gender considerations in traumatic stress studies is briefly described. Third, cultural distinctions and legal contexts shaping the understanding and inclusion of these concepts, with non-Western and low-to-middle income regions facing significant legal and ethical obstacles are highlighted. Methodological challenges including measurement, recruitment, and statistical modelling are discussed, followed by recommendations including participatory approaches that involve members of the community, including sexual and gender minority individuals, as possible, throughout the research process, conducting risk analyses, employing sensitive quantitative and qualitative methods, and ensuring clear reporting and participant protection. To conclude, with this letter, we hope to instigate dialogue and foster innovative approaches to incorporating sex and gender considerations in cross-cultural studies of traumatic stress. Addressing these considerations is essential for ethical, meaningful research that respects and safeguards diverse experiences.

## HIGHLIGHTS

- Sex and gender considerations are essential to further our understanding of trauma and its consequences.
- Considering sex and gender poses significant legal, ethical, and methodological challenges in cross-cultural studies involving non-Western and low-to-middle-income countries.
- Proposed mitigation strategies include using participatory approaches that involve members of the community, including sexual and gender minority individuals, as possible in research teams, conducting risk analyses, sensitive quantitative and qualitative methods, and clear reporting.

## Abstract (es)

Después de la 1^o^ Conferencia de la Colaboración Global sobre Estrés Traumático, el consorcio se comprometió a integrar sistemáticamente las consideraciones de sexo y género en sus iniciativas, lo cual se alinea con la Política de Género de la Revista Europea de Psicotraumatología. Esta iniciativa es crucial para comprender los complejos impactos del trauma, pero también presenta desafíos importantes en la investigación transcultural. Esta carta, coescrita por investigadores de todo el mundo, describe estos desafíos y propone estrategias de mitigación. Primero, se proporcionan definiciones de sexo y género desde la perspectiva Occidental, a la vez que se reconocen las diferencias culturales en estos conceptos. Segundo, se describe brevemente la relevancia de integrar consideraciones de sexo y género en los estudios sobre estrés traumático. (3) Se destacan las distinciones culturales y los contextos legales que moldean la comprensión e inclusión de estos conceptos, con regiones no occidentales y de ingresos bajos a medios que enfrentan obstáculos legales y éticos significativos. Se discuten los desafíos metodológicos, incluyendo la medición, el reclutamiento y el modelo estadístico, seguido de recomendaciones que incluyen enfoques participativos que involucran a los miembros de la comunidad, incluidas personas de minorías sexuales y de género, en la medida de lo posible, durante todo el proceso de investigación, realizando análisis de riesgo, empleando métodos cuantitativos y cualitativos sensibles, y garantizando informes claros y la protección de los participantes. Para concluir, con esta carta, esperamos iniciar un diálogo y fomentar enfoques innovadores para incorporar consideraciones de sexo y género en los estudios transculturales sobre el estrés traumático. Abordar estas consideraciones es esencial para llevar a cabo investigaciones éticas y significativas que respeten y protejan diversas experiencias.

## 1. Introduction

After the 1st Conference of the Global Collaboration on Traumatic Stress in December 2023, the consortium, comprised of clinicians, researchers, and policymakers from across the globe, resolved to systematically integrate sex and gender considerations in their research projects. This decision, outlined on the organisation's website (https://www.global-psychotrauma.net/sex-gender), echoes the Gender Policy enacted by the *European Journal of Psychotraumatology* (EJPT) in 2016. While crucial for enhancing our comprehension of trauma's intricate ramifications, the deliberate inclusion of sex and gender elements presents distinct challenges, particularly in the context of cross-cultural research. This letter to the editor endeavours to succinctly outline these obstacles and propose potential remedies, with the overarching goal of initiating dialogue and fostering innovative, ethical approaches to incorporating sex and gender considerations in cross-cultural studies of traumatic stress.

## 2. Defining sex and gender

In the Western world, there has been increasing emphasis placed on the importance of distinguishing between sex and gender in psychological research to better understand their distinct influence on study outcomes. While we recognise that this distinction may not apply worldwide, we will define these concepts from a Western perspective as a starting point, using the Canadian Institutes of Health Research (CIHR) definitions.

Although the terms sex and gender are often used interchangeably, they represent two separate, but related constructs. Sex refers to the collection of biological and physiological traits a person is born with. It describes the ways in which a person’s body is organised and functions, including genetic composition, hormone fluctuations and reproductive anatomy (CIHR, 2023). Sex is typically assigned at or before birth, based on external reproductive anatomy (i.e. male, female, or intersex) (CIHR, 2023; Intersex Society of North America, nd). Gender, on the other hand, reaches beyond the binary to describe the diverse ways people perceive, understand, and express themselves in relation to socially constructed roles and norms (CIHR, 2023). The broad spectrum of gender identity includes people whose identity aligns with their sex-at-birth (i.e. cisgender) and those who experience their gender as different from the sex they were assigned at birth (i.e. gender diverse). Sexual orientation and sexuality, are related constructs that will not be discussed further in this letter, but refer to an individual’s experience of sexual, emotional, and romantic attraction to people of same or another gender (Little, 2016).

## 3. Sex and gender in history and culture

Important social and cultural distinctions are present in the understanding, definitions, and language surrounding sex and gender. The term sex has historically and continues to describe aspects of biology, whereas the definition of gender has evolved alongside the social and historical context. Prior to the 1950s, the term gender referred solely to grammatical categories but began to take on a new meaning when feminist scholars adopted it to emphasise the distinct sociocultural roles of men and women in the home and society at large (Money, 1994). This perspective of sex and gender is still present in many countries around the world, such as countries in sub-Saharan Africa, Southeast Asia, and in the Middle East North Africa (MENA) region (International Lesbian, Gay, Bisexual, Trans and Intersex Association, 2019). For example, Middle Eastern countries are predominantly non-secular, resulting in gender definitions that are closely aligned with religious binary views on gender (Abouchedid, 2007; AbuKhalil, 1997). Furthermore, some languages may not have words to distinguish between concepts of sex and gender as they are understood in the Western context. However, it would be remiss not to acknowledge that several indigenous communities worldwide have historically embraced the fluidity of gender, an understanding that has been lost due to colonisation (Fiani & Serpe, 2020; Robinson, 2020; Picq, 2019).

## 4. Why are sex and gender important in traumatic stress studies?

Traumatic stress research has documented sex and gender differences in many aspects of the trauma experience, from the type of events experienced to the responses to treatment (Langeland & Olff, 2024). For instance, men have been found to experience more war-related traumas, accidental injuries, serious illnesses, physical assaults, and terrorist attacks than women (Christiansen, 2023; Kimerling et al., 2018; Tolin & Foa, 2006). On the other hand, women are more likely than men to experience all forms of childhood maltreatment, sexual assault, rape, intimate partner violence, kidnapping, and stalking (Langeland & Olff, 2024; Kimerling et al., 2018; Tolin & Foa, 2006). Additionally, women are more likely than men to develop post-traumatic stress disorder (PTSD) symptoms and to develop them early in life (Christiansen, 2023; Kimerling et al., 2018; Tolin & Foa, 2006), however, the higher PTSD prevalence in women appears to be less stable in longitudinal follow-ups (Haering et al., 2024). Interestingly, emerging evidence indicates that women and men may respond differently to psychotherapeutic interventions (Christiansen, 2023; Kimerling et al., 2018; Ascienzo et al., 2022). In terms of gender diversity and traumatic stress, transgender and gender-diverse participants report higher rates of lifetime exposure to traumatic experiences and current PTSD compared with cisgender participants (Bedford et al., 2023).

Few studies have examined potential mechanisms underlying sex and gender differences in an individual’s vulnerability to traumatic stress symptoms and response to evidence-based interventions, however, possible explanations include the types and patterns of exposure to traumatic events that differ among male, female, and gender-diverse individuals (Langeland & Olff, 2024; Kimerling et al., 2018). The heightened exposure of gender-diverse individuals to discrimination, stigma, and poverty may contribute to their heightened risk of presenting with post-traumatic distress (Keski-Rahkonen, 2023; Mark et al., 2019). For instance, research indicates that in the MENA regions, these populations often endure multiple and prolonged trauma experiences, including assaults, psychological abuse, blackmail, forced conversion therapy, and public shaming (Alessi et al., 2016; 2018). Broader social contexts discouraging males’ expression of distress may also partially explain these differences (Kimerling et al., 2018; Street & Dardis, 2018). Sex differences pertaining to differential biological susceptibility for male and female individuals may also be present (e.g. hormones, heritability) (Li & Graham, 2017; Duncan et al., 2018; Ravi et al., 2019), but all these hypotheses have yet to be supported with strong methodological designs allowing for the disaggregation of sex effects from gender effects. Such studies are even more strongly needed in non-Western and low-to-middle-income countries (LMIC), where exposure to trauma can be extremely high (Magruder et al., 2017).

## 5. Challenges related to sex and gender considerations in cross-cultural traumatic stress research and possible mitigation strategies

Key challenges related to the integration of sex and gender considerations in cross-cultural traumatic stress research including non-Western and LMIC are legal and ethical. A third category of challenges, methodological ones, may be equally potent everywhere in the world when conducting cross-cultural studies.

### 5.1. Legal challenges

Considering gender as a non-binary concept within non-Western and LMIC contexts involves significant legal challenges. The latest *Trans Legal Mapping Report* sheds light on these issues, particularly in sub-Saharan Africa, where despite some judicial advancements, persecutory criminal laws in many African states leave transgender and gender diverse individuals vulnerable to arrest, detention, harassment, and abuse by law enforcement (International Lesbian, Gay, Bisexual, Trans and Intersex Association, 2019). While certain countries explicitly criminalise diverse gender expressions, others indirectly subject transgender and gender diverse people to criminalisation through broader legal provisions such as public order, indecency laws, and criminalisation of consensual same-sex conduct (International lesbian, gay, bisexual, trans and intersex association, 2017). Similarly, in Asia, the latest report highlights instances of regression or stagnant progress, including leveraging laws prohibiting male homosexuality to target trans women, requirements for sterilisation of transitioning individuals, and the absence of legal gender recognition contributing to violence against trans individuals by law enforcement.

Latin American and Caribbean countries, as well as European regions exhibit varying degrees of recognition of trans and gender diverse rights, with some prohibiting name and sex changes entirely, while others allow these changes without surgical requirements and recognise gender categories beyond male and female. However, similar to Africa and Asia, some countries in these regions employ general legal provisions related to consensual same-sex and public morality, sex-work regulations, and police identity control laws, to discriminate against trans and gender diverse individuals. The report, and other sources, underscores a pervasive pattern of harassment, arrest, and detention of trans individuals by law enforcement without legal justification (Malta et al., 2019; International Lesbian, Gay, Bisexual, Trans and Intersex Association, 2019).

In North America, while Canada has made recent strides in recognising non-binary identities, these advancements face resistance from anti-trans activists, and accessibility remains a challenge for marginalised members of the trans community (DeGagne, 2021; International Lesbian, Gay, Bisexual, Trans and Intersex Association, 2019). Similarly, the United States’ treatment of trans and gender diverse individuals varies significantly partly based on the progressiveness or conservatism of individual states. Finally, Oceania also displays regional discrepancies, with Australia and New Zealand offering legal gender recognition in contrast to other parts of the region (International Lesbian, Gay, Bisexual, Trans and Intersex Association, 2017).

### 5.2. Ethical challenges

Considering these legal challenges, it becomes apparent that significant ethical hurdles confront the incorporation of sex and gender in cross-cultural traumatic stress research, particularly in non-Western and LMIC populations. Achieving a balance between ensuring participant safety and demonstrating respect poses a notable challenge (Cartwright & Nancarrow, 2022; Cameron & Stinson, 2019), especially as individuals from regions where gender diversity is not only unacknowledged but also potentially criminalised, either indirectly through secondary legal provisions or directly by law, may face risks in disclosing their non-binary gender identity. While the utilisation of binary gender inquiries may be perceived as disrespectful by transgender and gender diverse participants, non-binary inquiries could antagonise participants with more traditional views on gender.

In our experience conducting multi-country investigations, offering participants a choice other than male or female on sociodemographic questionnaires can be met with resistance from ethics review boards in regions that do not recognise gender diversity. Within these contexts, researchers are limited in the ways they can include sex and gender considerations in their studies and may be unable to disentangle their differential impact. Qualitative interviews may facilitate spontaneous disclosure of gender diverse identities provided questions are sensitively framed and interviewers convey openness regarding these topics; anonymous surveys may also facilitate disclosure. For self-reported questionnaires, inclusion of a query regarding sex assigned at birth could mitigate conflations between sex and gender in measurements. In instances where ethics committees restrict gender options to male and female, offering a ‘refuse to answer’ alternative or asking an open-ended question may serve as a tactful means of implicitly acknowledging gender diversity (Cartwright & Nancarrow, 2022). Importantly, researchers are encouraged to conduct a risk analysis before deciding on the inclusion of sex and gender considerations in their research; such an analysis may help determine if the benefits of this inclusion outweigh the risks.

### 5.3. Methodological challenges

Researchers wishing to consider sex and gender and disentangle their effects in cross-cultural traumatic stress research may also face important methodological challenges, notably in terms of measurement, recruitment, statistical modelling and qualitative analyses, and in sensitively and ethically reporting findings.

*Measuring* sex and gender across cultures and languages constitutes a major challenge. For instance, in Turkish and many other languages, the distinction between sex and gender is not as frequently used or understood compared to in English, and the same word is often used to refer to both. To address potential confusion about specific terms, researchers could provide a lay definition alongside questions about biological sex and gender identity to ensure accurate and consistent responses despite differences in understandings. When data are collected as part of an interview, it is essential that enumerators are trained, understand definitional nuances, and ensure participants' safety by adhering to principles of confidentiality, especially when working with vulnerable populations (e.g. refugees or other displaced groups, minority groups).

Measuring biological sex markers and gender identity in a way that allows these constructs to be disentangled may also prove difficult. The two-step measurement approach – involving first asking a question about *sex assigned at birth* and appearing on the birth certificate (an imperfect but useful proxy for anatomical, genetic, and physiological sex traits) and second asking about *current gender* – has been proposed as a relatively simple way to measure both sex and gender (Committee on Measuring Sex, Gender Identity, and Sexual Orientation, Committee on National Statistics, Division of Behavioral and Social Sciences and Education, National Academies of Sciences, Engineering, and Medicine, 2022). Yet, translating these straightforward questions and response options into some languages may pose challenges, underscoring the need for culturally sensitive adaptations.

*Recruitment* may also prove challenging, especially of trans and gender-diverse individuals who represent an often stigmatised, sometimes illegal, minority of the general population. In the North American and Western European contexts, recruiting male participants is also harder than recruiting female participants who are more willing to enter research studies (Ryan et al., 2019). Including sexual and gender minorities from the region where the study takes place on the research team, and/or collaborating with organisations that support this population may improve recruitment efforts. This should be undertaken with consideration of the potential risks that participation may entail in different contexts.

In quantitative research, *statistical modelling* issues also arise when considering sex and gender and aiming to disentangle their unique effects on outcomes. Statistical power is often an issue, especially when studying infrequent phenomena (e.g. posttraumatic stress disorder) and their intersection with minority identities. Not recruiting sufficient male, female, and gender-diverse participants will result in a lack of statistical power, impairing researchers’ ability to conduct disaggregated or comparative analyses. Non-parametric statistical analyses could be a solution to circumvent the stringent assumptions usually associated with parametric testing. Combining various categories of gender identity to attain a sufficient sample size in each cell may be another avenue but is not without limitations, and could be perceived negatively by some study participants and reviewers as it may obscure the unique experiences of subgroups of minoritized individuals and disregard the diversity among these individuals. Targeted recruitment efforts for each category of sex and gender, with specific strategies that have proved effective with these populations may be another option. The CIHR provides many resources to help researchers better disentangle sex from gender, even when using data already collected without gender measures (see https://cihr-irsc.gc.ca/e/50836.html).

Qualitative designs may also be considered, as in-depth interviews and focus groups can be used to explore the lived experiences of gender diverse individuals in detail. These methods allow for a richer understanding of personal and contextual factors influencing their experience of traumatic stress. Such methods can provide nuanced insights that quantitative methods might miss, especially regarding how socio-cultural factors intersect with sex and gender. Where possible, it is recommended to engage with gender-diverse individuals as co-researchers, which can be an empowering experience for marginalised groups, and ensures that their perspectives and needs are directly addressed.

Finally, *reporting* may also pose some challenges. To ensure the replicability of findings and facilitate a common understanding of sex and gender constructs that were measured in various cultural contexts, researchers should provide clear definitions in their dissemination material. A statement from the authors related to their positionality on sex and gender, as often used in qualitative research, may also prove effective in enhancing transparency in reporting. Additionally, ensuring participant safety by maintaining participant confidentiality and anonymity, especially in studies with few participants with unique characteristics (e.g. uncommon gender identity in their country, specific experiences of trauma) is crucial, but may also prove difficult in some circumstances. Ensuring studies on sex, gender, and traumatic stress are not used inappropriately to justify further discrimination and stigmatisation (e.g. of women or gender-diverse individuals) is essential. To minimise these risks, researchers should report their findings with a detailed contextualisation and be explicit about study limitations and applicability. Monitoring how their research is relayed publicly may also prove helpful, as well as taking responsibility for knowledge transfer efforts to control the message and ensure the appropriate nuances are provided to contextualise the findings. The inclusion of people from the population being studied when preparing publications and knowledge transfer material is also advised.

## 6. Conclusion

In conclusion, addressing sex and gender considerations in cross-cultural traumatic stress research in non-Western and LMIC contexts is crucial. However, legal aspects, cultural norms, and language affect the feasibility and safety of including sex, gender, and non-binary categories in research. Ensuring cultural sensitivity and the acceptability of research protocols is essential. Involving individuals from the regions of data collection on the research team, from planning to dissemination, is vital. Protecting participants, especially regarding gender diversity, is paramount. Prioritising these factors ensures ethical and meaningful research that respects and safeguards diverse experiences.

## Data Availability

There is no data related to this letter to the editor.
